# Integration of federated learning with IoT for smart cities applications, challenges, and solutions

**DOI:** 10.7717/peerj-cs.1657

**Published:** 2023-12-06

**Authors:** Yazeed Yasin Ghadi, Tehseen Mazhar, Syed Faisal Abbas Shah, Inayatul Haq, Wasim Ahmad, Khmaies Ouahada, Habib Hamam

**Affiliations:** 1 Department of Computer Science and Software Engineering, Al Ain University, Abu Dhabi, UAE; 2 Department of Computer Science, Virtual University of Pakistan, Lahore, Punjab, Pakistan; 3 School of Electrical and Information Engineering, Zhengzhou University, Zhengzhou, Henan, China; 4 Department of Computer Science and Information Technology, University of Malakand, Chakdara, Dir, Pakistan; 5 School of Electrical Engineering, Department of Electrical and Electronic Engineering Science, University of Johannesburg, Johannesburg, South Africa; 6 Commune d’Akanda, International Institute of Technology and Management, BP Libreville, Estuaire, Gabon; 7 Faculty of Engineering, University of Moncton, Moncton, New Brunswick, Canada; 8 College of Computer Science and Engineering, University of Ha’il, Ha’il, Saudi Arabia; 9 Production & Skills Development, Spectrum of Knowledge Production & Skills Development, Sfax, Tunisia

**Keywords:** AI, Smart grid, Federated learning, Internet of things, Blockchain, Machine learning

## Abstract

In the past few years, privacy concerns have grown, making the financial models of businesses more vulnerable to attack. In many cases, it is hard to emphasize the importance of monitoring things in real-time with data from Internet of Things (IoT) devices. The people who make the IoT devices and those who use them face big problems when they try to use Artificial Intelligence (AI) techniques in real-world applications, where data must be collected and processed at a central location. Federated learning (FL) has made a decentralized, cooperative AI system that can be used by many IoT apps that use AI. It is possible because it can train AI on IoT devices that are spread out and do not need to share data. FL allows local models to be trained on local data and share their knowledge to improve a global model. Also, shared learning allows models from all over the world to be trained using data from all over the world. This article looks at the IoT in all of its forms, including “smart” businesses, “smart” cities, “smart” transportation, and “smart” healthcare. This study looks at the safety problems that the federated learning with IoT (FL-IoT) area has brought to market. This research is needed to explore because federated learning is a new technique, and a small amount of work is done on challenges faced during integration with IoT. This research also helps in the real world in such applications where encrypted data must be sent from one place to another. Researchers and graduate students are the audience of our article.

## Introduction

Nobody wants unauthorized access to their data, whether a business or an individual. For the training of machine learning (ML) algorithms, a large quantity of high-quality data is required (Eyke, Green & Jensen, 2020). Federated learning (FL) is a method of training AI without needing a centralized server. Instead, it collaborates between a server and many devices. To make this function, no data must be transmitted back and forth (Ahmadi et al., 2022). It allows the storage of personal data locally, which reduces the risk of security breaches. When training the ML algorithms, traditional ML uses all the training data stored in the central server (Shen et al., 2022). It has a few issues; sometimes, connectivity between the device and the central server can be slow. Unauthorized individuals can access personal information. FL is a way to train models across multiple decentralized edge devices (Imteaj et al., 2021).

A global share model makes it possible for several devices to learn together. It will only send the collected information of the model, such as parameters and results, to the cloud, but it will use the data on devices to update the model (Jintasuttisak, Edirisinghe & Elbattay, 2022). For example, the keyboard model predicts the next word but also protects the confidentiality of text messages. It is a decentralized ML strategy that keeps data in its original location and decreases the amount of hardware infrastructure requirements (Bonawitz et al., 2021).

The IoT is a collection of interconnected physical objects that can exchange and integrate data with other devices and systems through sensor devices, applications, and other technologies (Noura, Atiquzzaman & Gaedke, 2019). The Internet Protocol (IP) is used to identify computers on the Internet and enable users to communicate with one another.

The IoT aims to create devices that can self-report information and data regularly and improve efficiency (Beg et al., 2022). Data security is an important point, and FL is a powerful strategy for protecting user privacy. In the domain of IoT, the sensors are used to gather data that may include privacy-sensitive personal details. Many IoT smart devices use FL, a distributed ML technique that protects IoT security and privacy (Pang et al., 2020). In Nguyen et al. (2021a) and Nguyen et al. (2021b), it has been proposed that FL can be used in various IoT applications, including intelligent healthcare, transportation, and Unmanned Aircraft Vehicles (UAVs).  FL has made it easier to provide intelligent healthcare services by allowing ML models without requiring medical institutions to share patient data (Aldahiri, Alrashed & Hussain, 2021). FL with IoT still has a lot of challenges.

To provide better patient care, experts in the healthcare industry require reliable technology. Extensive and diverse data sets are needed to train an algorithm for clinical purposes. FL plays an essential role in this kind of scenario. Companies use internal information sources to train the same algorithm (Lee & Shin, 2020). The FL can potentially bring significant positive changes in the healthcare profession. Thus, if FL is successfully applied, it could lead the way for universal precision medicine by generating models that are impartial in their predictions (Antunes et al., 2022). FL can also verify that the algorithm is running correctly without compromising the patient’s safety. The fourth industrial revolution, 4.0, completely alters how companies produce, improve, and distribute their products. The IoT, cloud computing, AI, and ML are new technologies that integrate business operations (Helo & Hao, 2021). FL allows computer-based algorithms to monitor and operate machinery, robots, and vehicles (Vijayakumar et al., 2019).

FL enables multiple participants to develop a global predictive model cooperatively. This strategy has received much attention in large-scale architectures and generic loT applications (Safri et al., 2022). Using digital technologies results in a higher level of automation, predictive maintenance, and self-optimization of improved efficiency (Naveed, Anwar & Haq, 2021). FinTech companies use a wide range of technology to manage their financial operations (Mehrban et al., 2020). Businesses that rely on FinTech face several challenges. These challenges are about obtaining approval and legal agreements, questions raised regarding the safety of the data, and the amount of time and expenses involved in collecting and transmitting data between networks. FL provides an easy solution for ML that is both encrypted and distributed. It enables users to train ML together on distributed data without the requirement for data transmission at any point during the process (Liu et al., 2022). It can solve problems and provide answers to FinTech. FL clears the way for FinTech to minimize possible risks (Kawase et al., 2019). It develops innovative and forward-thinking approaches for the benefit of both its customers and the businesses. It is reasonable for both parties to have trust in one another.

**Figure 1:**
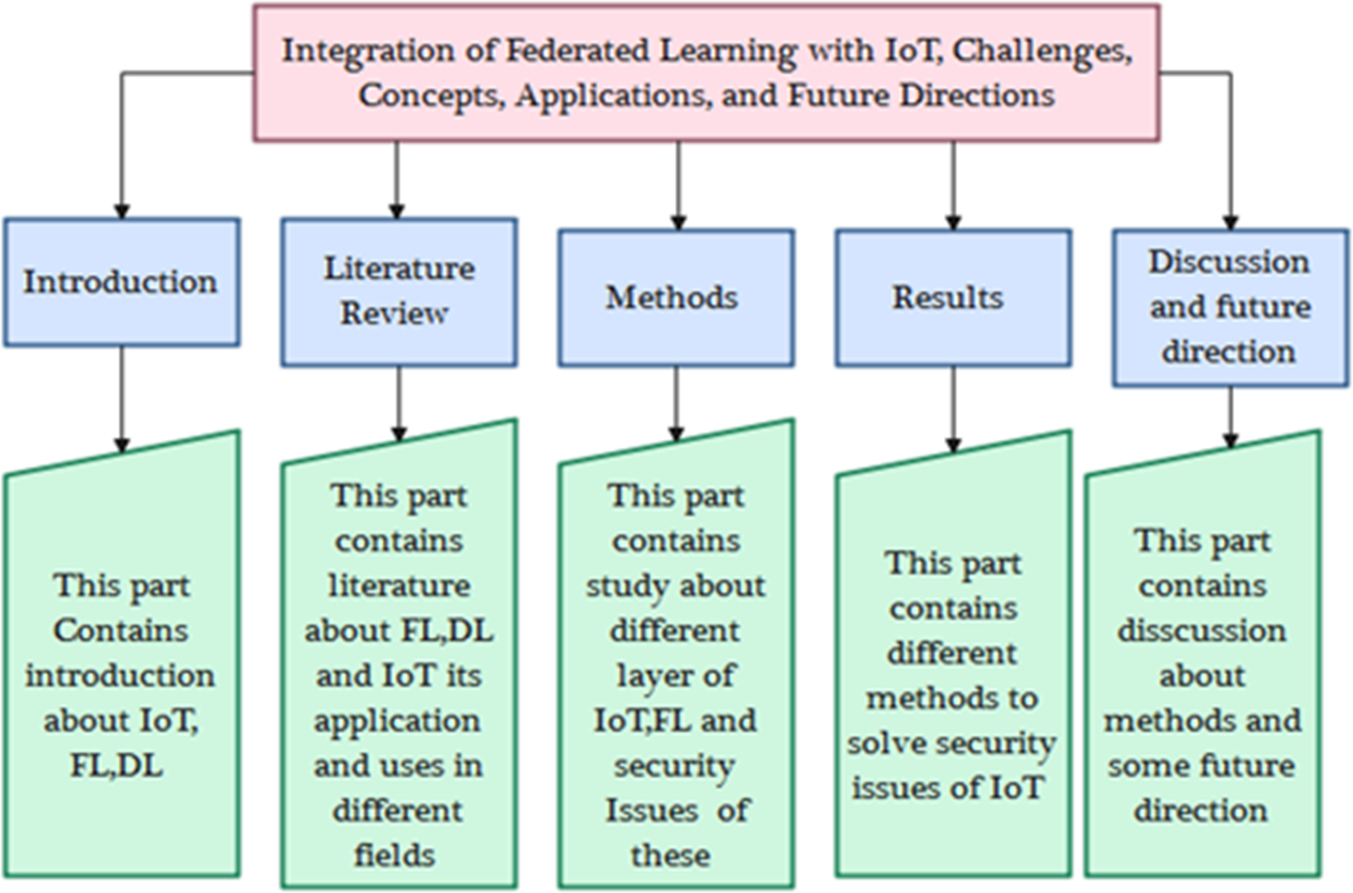
Article organization.

This study examines the obstacles and potential solutions to integrating FL and the Internet of Things (IoT). This statement underscores the significance of FL in protecting user privacy, enhancing the performance of models, facilitating adaptable scalability, and augmenting the quality of learning within the Internet of Things (IoT) networks. The study also discusses many obstacles faced by Federated Learning for the Internet of Things (FL-IoT). These challenges include resource management, Aggregation of updates, protection of privacy, security concerns, and issues related to learning and communication, standardized standards, and deploying machine learning capabilities on IoT sensors. This article identifies significant challenges and outlines potential future research lines. In ‘Literature Review’, we go through the most commonly used FL-IoT applications. ‘Methods and Techniques’ discusses the importance of FL and IoT and its challenges in detail, which is very important. ‘Results and Discussion’ represents the solutions and opportunities to FL-IoT Challenges. The conclusion and future work are described in ‘Conclusions and Future Work’. Figure 1 shows the article’s organization and Table 1 shows the list of abbreviations.

**Table 1:** The list of abbreviations.

**Abbreviations**	**Full form**
IoT	Internet of Things
AI	Artificial intelligence
FL	Federated learning
NLP	Natural language processing
DNN	Deep Neural Network
CNN	Convolutional neural network
D IoT	Defense Internet of Things
PKI	Public Key Infrastructure

## Literature Review

**Figure 2:**
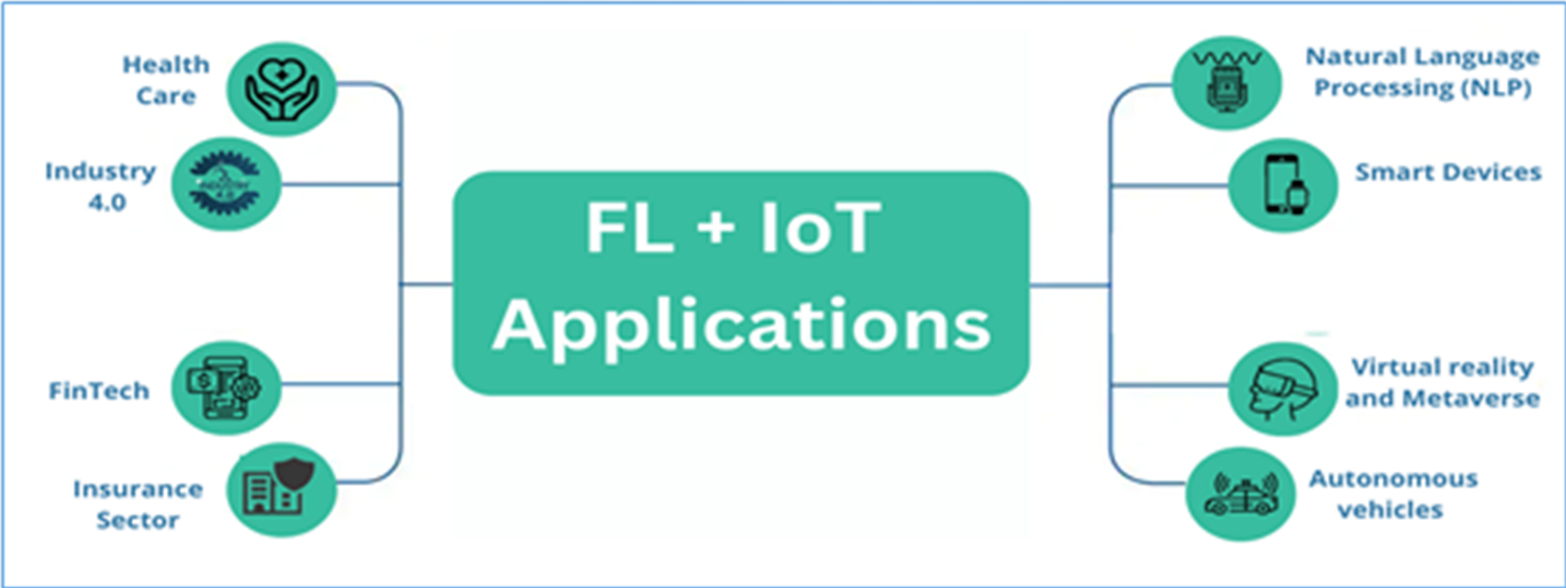
Application of FL for IoT.

FL allows insurance companies to train the model with its data and then upload the trained model’s output to the federation server because this method is secure and encrypted (Wang, Dang & Zhou, 2019). The federation server will integrate the model results to produce a superior model and send feedback to each contributor. It provides efficient distribution of model training and reduces the amount of money spent on data transfer and storage. FL aims to train ML algorithms with different data sets (Wahab et al., 2021). A company could identify its customers’ behaviors without violating the data. The deployment of FL helps to prevent illegal activities. The algorithms could use the data to guide training and decision-making (Zheng et al., 2022). Natural language processing is a part of AI that helps computers understand, analyze, and change human language. It contributes to improved comprehension of the semantics of human languages. A massive amount of data is required to train extremely accurate language models. This information is readily available from portable electronic devices such as mobile phones and tablets. Because the textual data gathered from each edge device contains user information, privacy concerns challenge central language learning models in this context. In Liu et al. (2021a) and Liu et al. (2021b), the authors presented that it is possible to construct natural language processing models using an FL framework. The amount of data also significantly increases as technology improves. In modern IoT networks, including wearable technology, driverless cars, and smart homes, sensors collect and process data in real-time (Bianchi et al., 2019). FL techniques enable the development of models that quickly adapt to these systems. Various organizations are now using FL. Without exchanging data, organizations train their algorithms on a variety of datasets. FL seeks to protect the information gathered through several routes and keeps valuable data near to reach (Liu et al., 2020a; Liu et al., 2020b; Liu et al., 2020c). Personalization can be accomplished with FL, and the functionality of IoT applications can be improved. Figure 2 shows the application of FL for IoT.

The future generation of the Internet is the metaverse. It will provide a fully connected, comprehensive, and engaging online 3D virtual reality experience that can be accessed on personal computers and other devices using virtual and augmented reality (Lee et al., 2021). Users will have control of their avatars in the metaverse, which allows them to communicate with virtual objects. The information gathered through IoT devices and servers connects the virtual and real worlds. FL is an attractive potential solution that could facilitate collaboration between the edge and the server, improving global performance and enhancing the metaverse’s level of security and privacy. Wearable technologies from millions of people can gather motion-tracking data that can be trained on local machines and put together on an FL server (Wang et al., 2022). Due to its real-time predictive abilities, FL is an essential part of the technology behind autonomous vehicles. Road and traffic conditions can be updated in real-time, which can be reflected in the data for continuous improvement and quick decision-making (Zhang et al., 2021). One possible benefit is that it will make driving more relaxing and secure. There is a lot of potential for using federated ML in the automotive industry.

FL may reduce the time spent training autonomous vehicles and improve the accuracy with which wheel and steering angle predictions are made (Zhang et al., 2021). The exponential increase in IoT devices has had a major and very far impact on people’s regular routines. AI dramatically affects how IoT devices are trained to use ML (Abdellah, Mahmood & Koucheryavy, 2020). The term “federated learning” refers to a type of “distributed ML” that is implemented in a wide variety of “smart devices” that are connected to the IoT. FL is ideal for edge networks, where each IoT device at the edge acts as an autonomous client (Lim et al., 2020). Sensors connected to the IoT can immediately collect information about users, including private information. FL provides an excellent way to protect user privacy by ensuring that raw data collected for each IoT device is not shared with other entities (Dash, Sharma & Ali, 2022). Each IoT device learns models in an FL environment to perform its task. When training in a federated way, the raw data never leaves the machines, and only the model updates are sent to the centralized server. This significantly reduces the potential for leaking sensitive information (Rahman et al., 2020).

FL reduces the requirement to transfer IoT data to the server and helps minimize interaction delays caused by information offloading. It helps preserve system resources during the information phase, like frequency and transmission power (Tran et al., 2019). There is not enough information to build a high-quality model from a single IoT device. As a result of FL’s model-building infrastructure, all devices can work together to produce superior models (Liu et al., 2020a; Liu et al., 2020b; Liu et al., 2020c). FL is an effective method for enhancing the model’s overall performance in FL that allows flexible scalability and limited computation power available at several IoT devices across various geographic locations (Zhang et al., 2021). Increasing the network’s capacity is challenging because combining all data on a single server depletes computer resources at the edges (Ferrer, Marquès & Jorba, 2019). FL can increase IoT networks’ flexibility without adding more load to central servers. Instead, it enables more devices to join the framework (Bhati, Chugh & Bhati, 2022). FL decreases the cost of communications, which is helpful for IoT networks with low bandwidth (Victor et al., 2022). FL can potentially increase the whole training phase’s convergence speed and attain higher learning accuracy rates. FL is employed in various IoT applications, such as UAVs, smart healthcare, and smart transportation (Nguyen et al., 2021a; Nguyen et al., 2021b).

FL has facilitated ML modeling to simplify delivering smart healthcare services. Health data providers like hospitals are not required to share health information when using FL. They localize the training of the AI model and transfer the trained parameters to the aggregator, enabling it to be used in global computation (Fadlullah & Kato, 2021). FL encourages collaborative healthcare environments, involves rapid patient assessment and treatment, and protects individual privacy (Banabilah et al., 2022). The advanced vehicular services offered by FL, including autonomous driving, road safety prediction, and vehicle identification, enhance driver training efficiency (Javed et al., 2022).

## Methods and Techniques

### Exclusion and inclusion

We searched several databases, including those managed by IEEE, Springer, Scopus, Google Scholar, ACM, Science Direct, and Wiley, using the search keywords “federated learning approaches” and “Internet of Things”. Articles were chosen for publication based on how well they addressed security, IoT integration, and FL categorization. The following journals contain these articles. After being chosen in the first step, some papers were studied in additional detail. The literature on FL-based methodologies was then produced to learn more about the operation of the IoT and how to keep it secure. We found a few more papers during our initial search but ignored them altogether. We only selected a few articles to review to get a sense of the current ML level and spot any holes in the literature that would need to be filled if the study went further. However, the review did not take any of the additional information into account.

### Research questions

The following research questions are in this study

What FL and IoT challenges are faced during integration with organizations like smart businesses, cities, transportation, and healthcare?

What are the solutions and opportunities for these problems?

### FL_IOT importance

The increasing intelligence that the IoT delivers to apps, businesses, gadgets, and industries significantly impacts daily lives. AI is anticipated to impact ML training performed on IoT devices considerably. The term “FL” refers to a type of “distributed ML” that is implemented in a wide variety of “smart devices” that are connected to the IoT. FL, being distributed and cooperative, is an excellent fit for edge networks in which each IoT device at the edge serves as an autonomous client. It is because FL was designed with these characteristics. FL effectively protects user privacy because raw data collected for each IoT device is not communicated to other parties. This is vital in IoT because sensors connected to the IoT can immediately capture data about users, including privacy-sensitive personal details (Sikder et al., 2021).

#### Data privacy for users

Each IoT device only acquires the minimum amount of knowledge required to carry out its recognition in a perfect FL situation. When training is federated, the raw data never leaves the devices, and only the model updates are sent to the centralized server. This significantly reduces the potential for leaking sensitive information (Liu et al., 2021a; Liu et al., 2021b).

### Improving model performance

A single IoT device will not have enough data to build a high-quality model by itself due to the constraints of personal devices. This is something to keep in mind. All of the IoT devices, when using the FL framework, have the capability of working together to train a high-quality model. This indicates that each person can benefit from learning data gathered by others in addition to their information, but without researching the private information of other participants. The FL could continually modify the local model in a time-varying manner, and the edge device was also competent in doing the same. Both of these capabilities were time-dependent. As a consequence, FL is an effective method for enhancing the model’s overall performance in a manner that is impossible for any individual device to achieve by itself (Bouacida & Mohapatra, 2021).

### Flexible scalability

FL is distributed; it can leverage the restricted computation power available at numerous IoT devices spread out over a wide range of geographic places similarly, enabling flexible scalability. FL can utilize these resources. The difficulty in growing the network’s capacity because centralizing all of the data on one server either loses the computer resources at the edges or tends to stress wireless networks (Wang et al., 2020). The size of the data of each individual gets to be extremely large as the capabilities of the edge device technology increase. FL can increase IoT networks’ flexibility without placing additional strain on central servers. This is achieved by encouraging more devices to sign up for the framework. There is no need within the FL framework for the lengthy transmission of raw data acquired by IoT devices. This decreases communications costs, which is highly helpful for IoT networks with low bandwidth (Sodin et al., 2021).

#### Enhanced learning quality

Using FL during the learning phase can speed up integration and improve learning outcomes. The FL methodological framework’s flexibility helps improve intelligent networks’ efficiency. Potential IoT uses for FL include UAVs, smart transportation, and smart healthcare. Giving patients smart healthcare services has become simpler because of the use of FL to simplify ML modeling. Hospitals and other healthcare facilities are not required to exchange patient information under FL. To enable it for global computing, they train the AI model locally and then communicate only its trained parameters to the aggregators. Florida promotes collaboration among medical facilities so that patients can receive faster diagnosis and treatment without compromising their right to privacy. FL has shown its ability to provide high-tech automobile services, including self-driving, forecasting traffic safety, more precise car identification during training, and increased privacy.

## Results and Discussion

### What FL and IoT challenges are faced during integration with organizations like smart businesses, cities, transportation, and healthcare?

**Figure 3:**
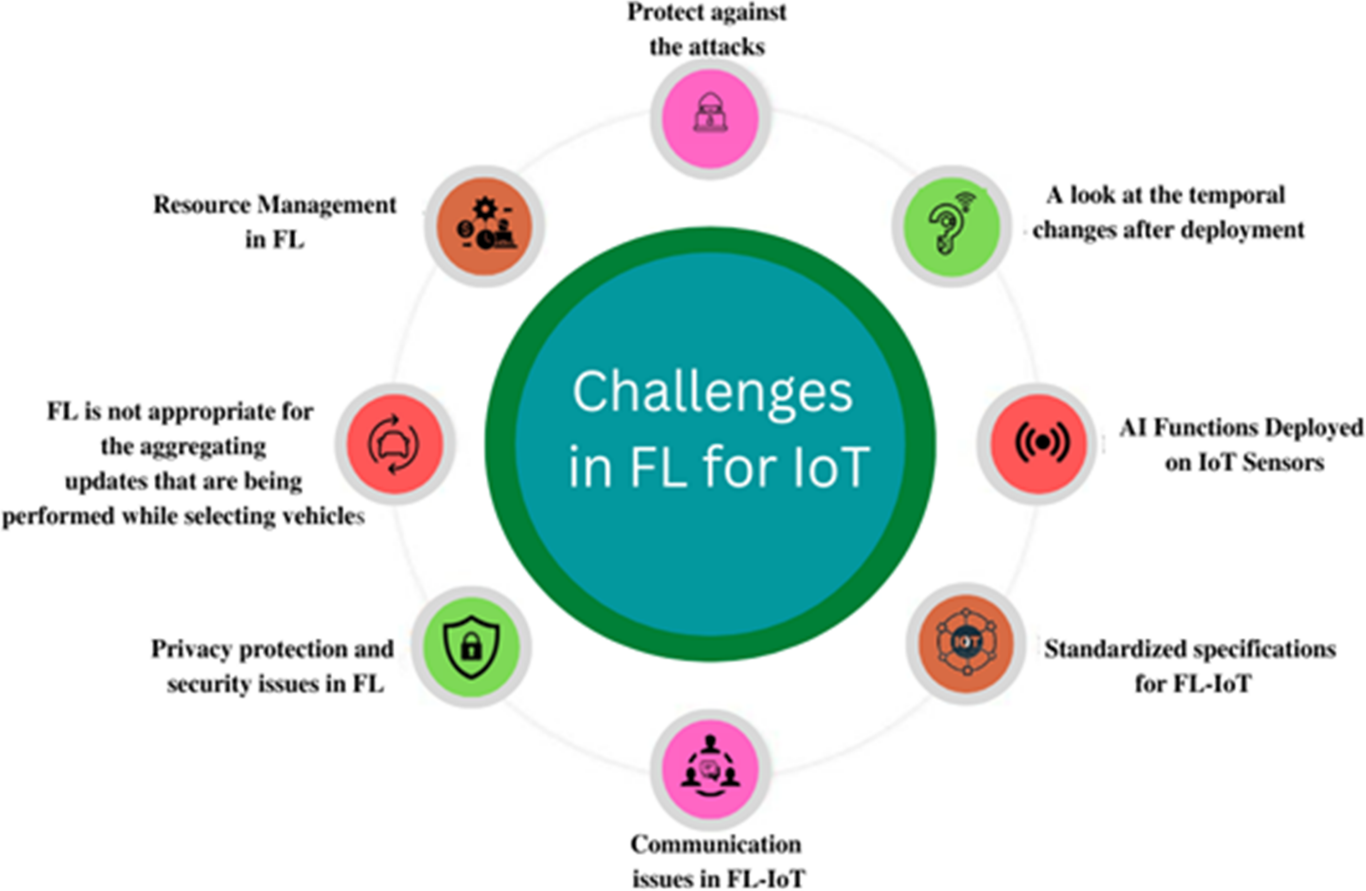
Challenges in FL-IoT.

FL is a relatively new field that has already made important contributions but also faces significant challenges. The following are key issues associated with FL (Li et al., 2020). These challenges prevent FL from being deployed on billions of IoT devices (Bouacida & Mohapatra, 2021). Figure 3 and Table 2 show the challenges in FL-IoT.

**Table 2:** The challenges in FL-IoT.

**SR. #**	**Challenges**	**The function of FL with IoT**	**Description**	**References**
1	Resource Management in FL	Resource management is used in neural networks to use computational resources effectively.	Specific IoT devices have limited computational capabilities, and the required resources for FL training are not always achieved.	Tsukada, Kondo & Matsutani (2020)
2	Updates Aggregations	The server must quickly collect and update information for vehicles and IoT devices.	Blockchain technology solves all of these challenges because it has decentralized storage and continued maintenance of the FL model.	Ali, Karimipour & Tariq (2021)
3	Privacy protection and security issues in FL	IoT needs a secure FL system with the highest possible protection for gathering data.	The central server can corrupt after a few iterations; hackers can steal the data to generate the training gradients.	Ma et al. (2020)
4	Learning and communication issues in FL-IoT.	High-quality model training in IoT networks requires an FL-based algorithm.	Due to the challenges posed by diverse data and the resources provided by distributed IoT devices, traditional FL algorithms have a slow convergence speed.	Nilssom et al. (2018)
5	Standardized specifications for FL-IoT.	Need a new set of Wi-Fi protocols to handle the heavy traffic loads of future IoT applications.	The deployment of FL-IoT ecosystems depends on other significant computing services, such as cloud computing. Network standards and aspects play an essential role in this deployment with fast network speed.	López-Pérez et al. (2019)
6	AI Functions Deployed on IoT Sensors.	To increase the accuracy of hardware learning, it is necessary to use a memory-effective on-device sensor learning system.	Some IoT sensors cannot take part in training a full-scale AI model due to hardware, memory, and power limitations.	Dhar et al. (2019)
7	Time variation after the deployment of the model.	The requirement for on-device model changes creates a new issue for IoT devices, which often have limited functional capacities.	The necessity for on-device model updates introduces a new challenge for the IoT devices, which frequently have limited capabilities in terms of their functionality.	Liang et al. (2020)
8	Protect against the attacks of the adversary.	IoT devices are easy targets of phishing, identity fraud, and distributed denial of services (DDoS).	The IoT system must implement a simple security protocol to identify malicious devices.	Salim, Rathore & Park (2020)
9	Relation with accessible resources.	Depending on the characteristics of the feature, IoT configurations and data collected by various devices could differ significantly.	An IoT FL framework should be able to adapt data and computation load among multiple devices based on the resources available to those devices.	Saeed, Ozcelebi & Lukkien (2019)

#### Resource management in FL

Federated learning has been investigated as a potentially useful option to train machine learning models at the network edge in a way that does not involve exchanging private user data. Because of the limited resources at the edge, new solutions need to be developed to maximize the use of the available software and hardware resources. It is necessary because the previously known solutions did not concentrate on resource management for the network edge, which is especially important for federated learning (Trindade, Bittencourt & Da Fonseca, 2022). To achieve updates on the server, all IoT devices need computing and storage resources. However, it is not always satisfied due to the limited computing power of IoT devices. A significant delay can occur at the server (Tsukada, Kondo & Matsutani, 2020).

#### Aggregation updates in FL-IoT

When moving data between networks, one of the most critical aspects of network security is ensuring that each device in the network has been properly authorized. Previously, the identification was based on public key infrastructure (PKI), which requires that each device in a system exchange its own private encrypted identity message to the local authentication center (LAC) (Akhter et al., 2021). The currently available privacy frameworks, such as the differential privacy framework (Caldas et al., 2018), are insufficient to resolve the users’ privacy.

#### Privacy protection and security issues in FL-IoT

FL can protect users’ privacy for the IoT systems that are distributed. FL still has several security and privacy flaws, and these challenges exist on both the client and server sides of the learning process (Lyu, Yu & Yang, 2020). Communication constraints, poisoning, and backdoor operations are currently the most specific security issues; inferential attacks are the most important to the privacy of FL. Future research must make FL adaptable in real-world situations (Mothukuri et al., 2021).

#### Learning and communication issues in FL-IoT

The major challenge in FL-IoT is the limited integration of learning and communication. Autonomous driving technology is making its way into regular vehicles with the advancement of vehicular IoT. A reliable self-driving system requires a real-time connection with a multi-access communication environment (Yarradoddi & Gadekallu, 2022). Spatial and temporal variations of the vehicular environment necessitate an intelligent solution that can adapt to the changing climate. The typical centralized-over-cloud solution requires the driving system to send a considerable quantity of raw data to the server, which could result in privacy leakage (Zhang et al., 2022).

#### Standardized specifications for FL-IoT

Future intelligent networks will include vertical FL-IoT use cases. Federated learning (FL) is a cutting-edge artificial intelligence approach. For this purpose, existing mobile network architecture must be amended (Shaheen et al., 2022). Network standards and elements like cloud analysis servers and edge-IoT protocols are essential, and these components can contribute to the completion of FL-IoT ecosystems (Ray, Dash & De, 2019). Unprocessed information is stored in advanced technology by a secret confidentiality service, which incorporates machine learning (ML) training while removing data connections (Alam & Gupta, 2022). There is a need for a developed system to improve the effectiveness of advanced learning systems.

#### AI functions deployed on IoT sensors

Internet of Things sensors are limited in training a comprehensive AI model due to the hardware, storage, and energy resources. Powerful machine learning algorithms typically require significant storage and power to train models. The main challenge is finding out the energy usage problem in FL-IoT systems. Improving the use of AI hardware on IoT sensors is an important task (Dhar et al., 2021).

#### Temporal changes after deployment

IoT sensing devices continuously collect updated information to upgrade the models for continuous learning. This data also improves the user experience (Tang et al., 2019). To continue offering services, it is also necessary to maintain training data on updated models, which presents a new issue for resource-constrained IoT devices. Most IoT devices have a limited amount of memory and memory resources. To overcome this problem, the requirement for a lightweight machine learning model can significantly reduce the memory needed for on-device training (Peltonen et al., 2020).

#### Protect against the attacks of the adversary

IoT devices are the targets for attackers, such as distributed denial of service (DDoS) (Parra et al., 2020). The traffic volume of IoT-based Attacks is increasing. Security updates can stop these attacks, but most IoT devices do not have sufficient processing power (Rizvi et al., 2018). The IoT system must be able to identify malicious devices that can destroy the training model. Implementing a lightweight security procedure and detecting malfunctioning devices in the IoT system is a solution (Jeon, Park & Jeong, 2020).

#### Relation to limited on-device resources

The existing machine learning models, particularly deep neural networks, demand significant computational power (Ren, Deng & Xie, 2022). Customized and specialized hardware is one way to speed up the training process for edge machine-learning applications. Edge devices have limited resources for memory storage, computational power, and information access (Qian et al., 2019). Recent neural network architectures are becoming increasingly complex. One of the most significant issues associated with neural network processing models is minimizing the number of memory accesses (Gobieski, Lucia & Beckmann, 2019). Table 2 shows an overview and summary of FL-IoT challenges in FL with IoT in Organizations.

### What are the solutions and opportunities for these problems?

**Figure 4:**
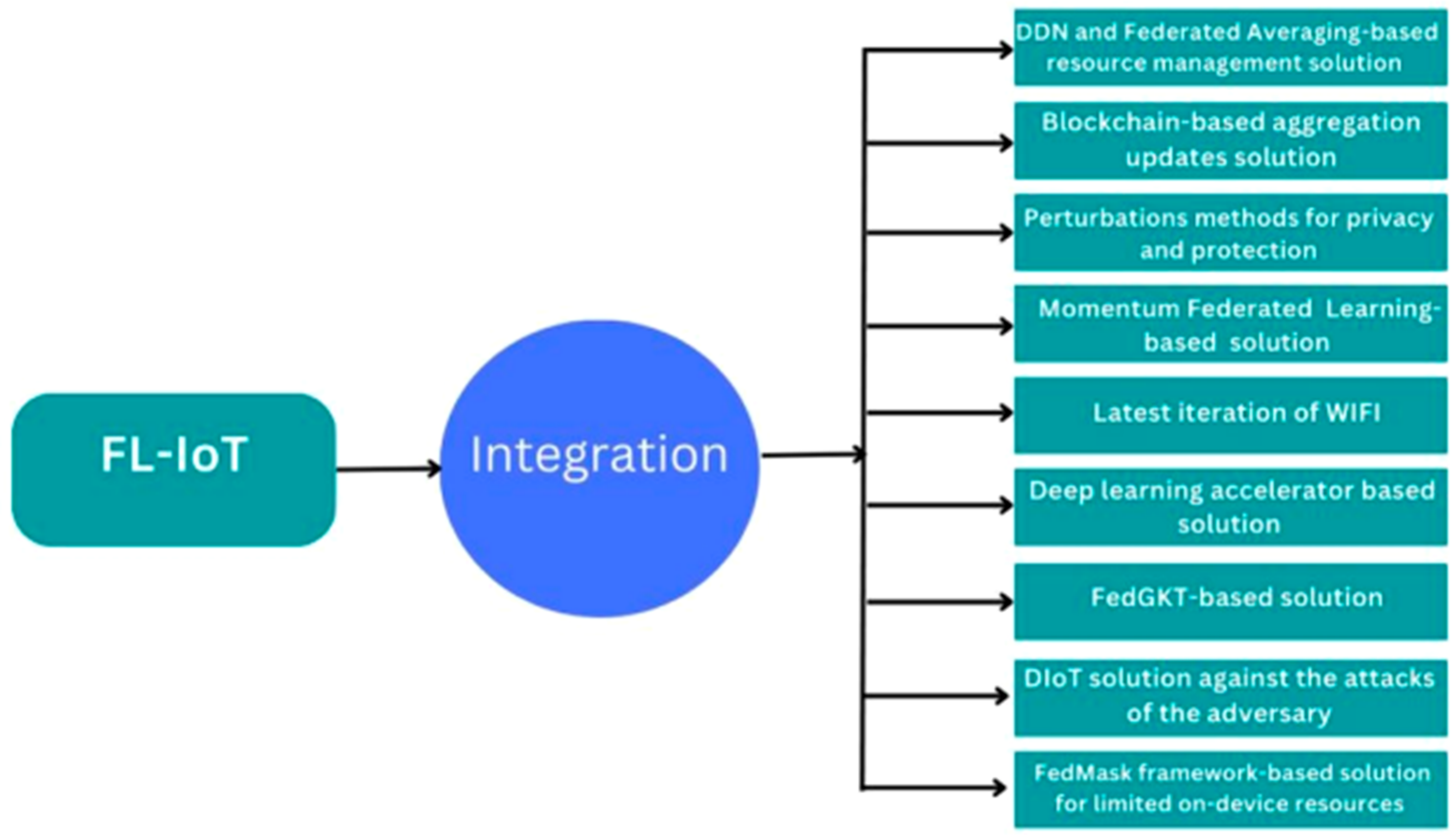
Integration of FL-IoT with solution models.

FL is a very emerging field with significant contributions but faces substantial challenges. New concepts and strategies are required to solve these challenges in the following subsections, the most popular solutions models that, when integrated with FL-IoT, may solve the challenges listed above. Integrating different solution models to FL-IoT can speed up the work and reduce the risks. The integration is presented in Fig. 4. Below, we have listed and explained all the important solution models in resolving issues in federated learning and IoT devices. Figure 4 and Table 3 show the integration of FL-IoT with solution models.

**Table 3:** The integration of FL-IoT with solution models.

**Challenges**	**Solution models**	**Models description**	**Role of models in IoT**	**References**
Resource management in FL	Train neural networks using an FL architecture that considers the resources available on mobile platforms.	This architecture takes into consideration information regarding the available computational resources.	A soft training method is included to speed up IoT devices.	Xu et al. (2019)
	DNN architecture for mobile devices training.	This model can speed up the learning process.	It can speed up the running time and save memory resources.	Li, Wang & Kong (2018)
	Federated wireless edge network learning resource management.	Algorithms are provided in this model to maximize efficiency based on the relevance of the data and the effectiveness of the correlating processing and transmission resources.	Improve the training’s accuracy, consistency, and speed of convergence.	Balakrishnan et al. (2020)
Vehicles aggregation updates in FL-IoT	A solution to privacy problems in vehicles using encryption.	Information that has been most recently updated should be shared between global models and localized models.	Identifies the privacy concerns generated by the IoT.	(DOH and CHAE)
Privacy protection and security issues in FL	We are establishing composition theorems with complex mathematical formulations and perturbations methods.	Differential privacy techniques can be employed to protect training datasets from being hacked.	Prevents unauthorized access to the data in IoT devices.	Ma et al. (2020)
	A safe and secure aggregation method used in FL-based systems.	The goal is to provide the highest level of protection to unauthenticated network conditions.	Use encryptions in IoT devices for local changes.	Bonawitz et al. (2016)
Learning and communication issues in FL-IoT.	A fresh optimization approach for FL-based IoT networks.	This method can successfully train high-quality models, increasing communication effectiveness.	Errors accumulate in IoT devices.	Rothchild et al. (2020)
	Momentum-based FL.	Increase the rate at which FL algorithms arrive at solutions.	Minimize the loss of functions in IoT devices.	Liu et al. (2020a), Liu et al. (2020b) and Liu et al. (2020c)
Standardized specifications for FL-IoT.	Open Platform Communication Global Infrastructure.	Edge-IoT services are intended to be supported.	Implementation in edge-IoT settings.	Ray, Dash & De (2019)
	The latest iteration of Wi-Fi technology.	In the 5G and 6G eras, this technology will be capable of meeting the peak throughput needs that emerging applications for the IoT will establish.	Intelligent IoT services at the edge networks.	López-Pérez et al. (2019)
AI Functions Deployed on IoT Sensors.	A deep learning accelerator.	It is based on software that facilitates AI training on mobile devices.	Maximize using hardware resources for training data without impacting overall performance reliability.	Lane et al. (2016)
	A lightweight model training method.	This strategy makes use of the idea of model output exchange rather than the concept of parametric exchange.	It can solve communication latency issues in IoT devices.	Itahara et al. (2020)
Temporal changes after deployment	FedGKT method.	It is possible that using this strategy will reduce the number of trained memories that is necessary for efficient on-device learning.	Several small CNN models can transfer knowledge from IoT devices to a more prominent CNN stored on the cloud.	He, Annavaram & Avestimehr (2020)
Protect against the attacks of the adversary	DIoT solution.	Apply an FL approach to anomaly detection-based vulnerability scanning in the gateway.	Scan the gateways to IoT systems instead of storing data locally.	Nguyen et al. (2019)
Limited on-device Resources	Fed Mask solution.	A framework that is efficient in terms of processing and communication.	IoT devices can build a sparse model with decreased computing cost and memory footprint.	Li et al. (2021)

#### DDN and federated averaging-based resource management solution for FL-IoT

Deep learning models like DNN require a considerable amount of CPU frequency to complete training tasks; it will be a good strategy to train the model directly on IoT devices (Jagmohan et al., 2018). In FL, the DNN models are distributed and taught on local machines. After that, the local parameters of the model are periodically aggregated in a centralized node to update the global model using an algorithm called federated averaging (FedAvg) (McMahan et al., 2017). Several strategies have been developed to simplify resource management in on-device FL training. To resource management problems, importance sampling and rank ordering-based algorithms were developed. These algorithms allow for the prioritizing of clients based on the resources they have accessible. The paper also presents algorithms for data-importance and computation communication-aware resources to optimize the efficiency of training convergence rate (Balakrishnan et al., 2020).

#### Blockchain-based aggregation updates solution for FL-IoT

FL solves privacy concerns to the maximum level. Blockchain technology is used in these scenarios to resolve the issues (Sharma, Park & Cho, 2020). In Unal et al. (2021), a secure architecture for FL in big data analytics services for IoT that uses blockchain technology was presented. The author provided an architecture that uses FL to train the model locally and then transmits the encrypted model to an edge-based artificial intelligence service. To provide accurate insights and predictions, the AI service compiles all of the regional models into one comprehensive model.

#### Perturbations methods for privacy and protection in FL-IoT

If a protection mechanism is not working, FL will act as a privacy and security firewall in intelligent IoT systems (Arisdakessian et al., 2022). By establishing composition theorems with complex mathematical formulations, perturbation methods such as differential privacy and dummies can be used to maintain the integrity of training datasets from being hacked. It is done to protect training data and hide personal information from external threats while guaranteeing convergence (Yin, Zhu & Hu, 2021). The findings of the study (Bonawitz et al., 2016) imply that there is a secure aggregation method for safe FL systems. It provides the best protection possible against server-mediated, unauthenticated network conditions. In Zhao et al. (2021), the FL scheme that protects users’ privacy for the extensive industrial data is represented. Differential privacy is applied *via* a Gaussian technique to shared parameters to ensure high user privacy protection.

#### Momentum federated learning-based solution in FL-IoT

The authors (Rothchild et al., 2020) suggested a new optimization approach for FL-based IoT networks; improving communication effectiveness standards can be trained with this model. In Liu et al. (2020a); Liu et al. (2020b); Liu et al. (2020c), an entirely new design for FL is presented: momentum federated learning. It aims to improve the FL algorithms’ ability to join solutions quickly. It minimizes the loss of functions in IoT devices.

#### The use latest iteration of WIFI in FL-IoT

Wi-Fi has become an integral part of our daily lives and is also one of the essential communication protocols for the Internet of Things. The IEEE 802.11 technical group has recently started discussing the possibility of issuing the IEEE 802.11 Wi-Fi protocol with a higher processing capacity, the next version of the Wi-Fi standard (Restuccia, 2021). In the 5G and 6G era, these standards provide service in deploying FL-IoT edge networks with smart IoT devices.

#### Deep learning accelerator solution for FL-IoT sensors

The research by Lane et al. (2016) suggests a deep learning accelerator based on software that facilitates AI and DL training on mobile devices. The central concept is a set of diverse processors, such as graphics processing units (GPUs), in which each computing unit uses various computational resources to process different inference phases of deep learning models. In Itahara et al. (2020), the authors outline a simple model training method that addresses the transmission cost connected with on-device FL training.

#### FedGKT-based solution for FL-IoT

Federated group knowledge transfer (FedGKT) is a method that can potentially lower the amount of learning memory needed for efficient on-device learning. IoT devices can transfer data from small CNN models to a more prominent CNN on a cloud server. This strategy will reduce the trained memories necessary for efficient on-device learning.

#### DIoT solution against the attacks of the adversary in FL-IoT

The Internet of Things system has to be able to recognize malicious connections that have the potential to corrupt the training model. The solution is implementing a simple security procedure and identifying devices in the IoT system that aren’t functioning properly. FL can provide an approach to IoT devices as a distributed system that can protect devices (Ghimire & Rawat, 2022). Defense Internet of Things (DIoT) is the IoT device gateway solution that employs an FL method for anomalous detection-based security vulnerabilities (Nguyen et al., 2019).

#### FedMask framework-based solution for limited on-device resources of FL-IoT

One strategy for speeding the training process for edge applications that use machine learning is customized and specialized hardware. Edge devices have limited resources for memory capacity, processing power, and access to information. Federated Mask (FedMask) is an efficient framework in terms of processing and communication. When the Fed-Mask algorithm is implemented, each node can learn a sparse binary mask that is heterogeneous and structured (Li et al., 2021). IoT devices can create a sparse model using this approach, resulting in lower computational costs and a smaller memory footprint. Table 3 shows the solution models to the above challenges in FL-IoT for implementation in organizations.

## Conclusions and Future Work

ML is expanding and changing the technological world. FL applications are also facing challenges. Privacy is essential for everyone. Training an ML model using data stored on centralized servers is difficult. Different challenges and issues with FL-IoT integration are described in the manuscript mentioned above and improve businesses’ performance. We have shown the different challenges of federated ML with the IoT, which are described above. We have also shown solution models and methods which are solving these challenges. These solution models for FL-IoT are described in detail and mentioned in the above table and figure. There are still a lot of challenges in the field of FL with IoT. Despite the recent technological advancement, different models have made it possible to handle these challenges. Suppose we can find more solutions on time. In that case, it will be a tremendous driving factor for improving FL’s industrial and academic domains with the IoT.

##  Supplemental Information

10.7717/peerj-cs.1657/supp-1 Data S1 Raw data

## Additional Information and Declarations

### Data Availability

The following information was supplied regarding data availability: This is a review article, and no code or data was used.

## References

[ref-1] Abdellah AR, Mahmood OA, Koucheryavy A (2020). Delay prediction in IoT using machine learning approach.

[ref-2] Ahmadi M, Taghavirashidizadeh A, Javaheri D, Masoumian A, Ghoushchi SJ, Pourasad Y (2022). DQRE-SCnet: a novel hybrid approach for selecting users in federated learning with deep-Q-reinforcement learning based on spectral clustering. Journal of King Saud University-Computer and Information Sciences.

[ref-3] Akhter AS, Ahmed M, Shah AS, Anwar A, Zengin A (2021). A secured privacy-preserving multi-level blockchain framework for cluster based VANET. Sustainability.

[ref-4] Alam T, Gupta R (2022). Federated learning and its role in the privacy preservation of IoT devices. Future Internet.

[ref-5] Aldahiri A, Alrashed B, Hussain W (2021). Trends in using IoT with machine learning in health prediction system. Forecasting.

[ref-6] Ali M, Karimipour H, Tariq M (2021). Integration of blockchain and federated learning for Internet of Things: Recent advances and future challenges. Computers & Security.

[ref-7] Antunes RS, Costa CAndréda, Küderle A, Yari IA, Eskofier B (2022). Federated learning for healthcare: systematic review and architecture proposal. ACM Transactions on Intelligent Systems and Technology (TIST).

[ref-8] Arisdakessian S, Wahab OA, Mourad A, Otrok H, Guizani M (2022). A survey on iot intrusion detection: federated learning, game theory, social psychology and explainable ai as future directions. IEEE Internet of Things Journal.

[ref-9] Balakrishnan R, Akdeniz M, Dhakal S, Himayat N (2020). Resource management and fairness for federated learning over wireless edge networks.

[ref-10] Banabilah S, Aloqaily M, Alsayed E, Malik N, Jararweh Y (2022). Federated learning review: fundamentals, enabling technologies, and future applications. Information Processing & Management.

[ref-11] Beg S, Handa M, Shukla R, Rahman M, Almalki WH, Afzal O, Altamimi ASA (2022). Wearable smart devices in cancer diagnosis and remote clinical trial monitoring: transforming the healthcare applications. Drug Discovery Today.

[ref-12] Bhati NS, Chugh G, Bhati BS (2022). Federated machine learning with data mining in healthcare. Federated learning for IoT applications.

[ref-13] Bianchi V, Bassoli M, Lombardo G, Fornacciari P, Mordonini M, De Munari I (2019). IoT wearable sensor and deep learning: an integrated approach for personalized human activity recognition in a smart home environment. IEEE Internet of Things Journal.

[ref-14] Bonawitz K, Ivanov V, Kreuter B, Marcedone A, McMahan HB, Patel S, Ramage D, Segal A, Seth K (2016). Practical secure aggregation for federated learning on user-held data.

[ref-15] Bonawitz K, Kairouz P, McMahan B, Ramage D (2021). Federated learning and privacy: building privacy-preserving systems for machine learning and data science on decentralized data. Queue.

[ref-16] Bouacida N, Mohapatra P (2021). Vulnerabilities in federated learning. IEEE Access.

[ref-17] Caldas S, Duddu SMK, Wu P, Li T, Konečný J, McMahan HB, Smith V, Talwalkar A (2018). Leaf: a benchmark for federated settings.

[ref-18] Dash B, Sharma P, Ali A (2022). Federated learning for privacy-preserving: a review of PII data analysis in fintech. International Journal of Software Engineering & Applications.

[ref-19] Dhar S, Guo J, Liu J, Tripathi S, Kurup U, Shah M (2019). On-device machine learning: An algorithms and learning theory perspective.

[ref-20] Dhar S, Guo J, Liu J, Tripathi S, Kurup U, Shah M (2021). A survey of on-device machine learning: an algorithms and learning theory perspective. ACM Transactions on Internet of Things.

[ref-21] Eyke NS, Green WH, Jensen KF (2020). Iterative experimental design based on active machine learning reduces the experimental burden associated with reaction screening. Reaction Chemistry & Engineering.

[ref-22] Fadlullah ZM, Kato N (2021). On smart IoT remote sensing over integrated terrestrial-aerial-space networks: an asynchronous federated learning approach. IEEE Network.

[ref-23] Ferrer AJ, Marquès JM, Jorba J (2019). Towards the decentralised cloud: survey on approaches and challenges for mobile, ad hoc, and edge computing. ACM Computing Surveys (CSUR).

[ref-24] Ghimire B, Rawat DB (2022). Recent advances on federated learning for cybersecurity and cybersecurity for federated learning for internet of things. IEEE Internet of Things Journal.

[ref-25] Gobieski G, Lucia B, Beckmann N (2019). Intelligence beyond the edge: inference on intermittent embedded systems.

[ref-26] He C, Annavaram M, Avestimehr S (2020). Group knowledge transfer: Federated learning of large cnns at the edge. Advances in Neural Information Processing Systems.

[ref-27] Helo P, Hao Y (2021). Artificial intelligence in operations management and supply chain management: an exploratory case study. Production Planning & Control.

[ref-28] Imteaj A, Thakker U, Wang S, Li J, Amini MH (2021). A survey on federated learning for resource-constrained iot devices. IEEE Internet of Things Journal.

[ref-29] Itahara S, Nishio T, Koda Y, Morikura M, Yamamoto K (2020). Distillation-based semi-supervised federated learning for communication-efficient collaborative training with non-iid private data.

[ref-30] Jagmohan C, Seneviratne S, Hu Y, Misra A, Seneviratne A, Lee Y (2018). Breathing-based authentication on resource-constrained IoT devices using recurrent neural networks. Computer.

[ref-31] Javed AR, Hassan MA, Shahzad F, Ahmed W, Singh S, Baker T, Gadekallu TR (2022). Integration of blockchain technology and federated learning in vehicular (iot) networks: a comprehensive survey. Sensors.

[ref-32] Jeon J, Park JH, Jeong Y-S (2020). Dynamic analysis for IoT malware detection with convolution neural network model. IEEE Access.

[ref-33] Jintasuttisak T, Edirisinghe E, Elbattay A (2022). Deep neural network based date palm tree detection in drone imagery. Computers and Electronics in Agriculture.

[ref-34] Kawase R, Diana F, Czeladka M, Schüler M, Faust M (2019). Internet fraud: the case of account takeover in online marketplace.

[ref-35] Lane ND, Bhattacharya S, Georgiev P, Forlivesi C, Jiao L, Qendro L, Kawsar F (2016). Deepx: a software accelerator for low-power deep learning inference on mobile devices.

[ref-36] Lee L-H, Braud T, Zhou P, Wang L, Xu D, Lin Z, Kumar A, Bermejo C, Hui P (2021). All one needs to know about metaverse: a complete survey on technological singularity, virtual ecosystem, and research agenda.

[ref-37] Lee I, Shin YJ (2020). Machine learning for enterprises: applications, algorithm selection, and challenges. Business Horizons.

[ref-38] Li L, Fan Y, Tse M, Lin K-Y (2020). A review of applications in federated learning. Computers & Industrial Engineering.

[ref-39] Li A, Sun J, Zeng X, Zhang M, Li H, Chen Y (2021). Fedmask: joint computation and communication-efficient personalized federated learning *via* heterogeneous masking.

[ref-40] Li D, Wang X, Kong D (2018). Deeprebirth: Accelerating deep neural network execution on mobile devices. Proceedings of the AAAI Conference on Artificial Intelligence.

[ref-41] Liang F, Yu W, Liu X, Griffith D, Golmie N (2020). Toward edge-based deep learning in industrial Internet of Things. IEEE Internet of Things Journal.

[ref-42] Lim WYB, Luong NC, Hoang DT, Jiao Y, Liang Y-C, Yang Q, Niyato D, Miao C (2020). Federated learning in mobile edge networks: a comprehensive survey. IEEE Communications Surveys & Tutorials.

[ref-43] Liu W, Chen L, Chen Y, Zhang W (2020c). Accelerating federated learning *via* momentum gradient descent. IEEE Transactions on Parallel and Distributed Systems.

[ref-44] Liu JC, Goetz J, Sen S, Tewari A (2021b). Learning from others without sacrificing privacy: simulation comparing centralized and federated machine learning on mobile health data. JMIR mHealth and uHealth.

[ref-45] Liu M, Ho S, Wang M, Gao L, Jin Y, Zhang H (2021a). Federated learning meets natural language processing: a survey.

[ref-46] Liu J, Huang J, Zhou Y, Li X, Ji S, Xiong H, Dou D (2022). From distributed machine learning to federated learning: a survey. Knowledge and Information Systems.

[ref-47] Liu Y, James J, Kang J, Niyato D, Zhang S (2020a). Privacy-preserving traffic flow prediction: a federated learning approach. IEEE Internet of Things Journal.

[ref-48] Liu Y, Peng J, Kang J, Iliyasu AM, Niyato D, Abd El-Latif AA (2020b). A secure federated learning framework for 5G networks. IEEE Wireless Communications.

[ref-49] López-Pérez D, Garcia-Rodriguez A, Galati-Giordano L, Kasslin M, Doppler K (2019). IEEE 802.11 be extremely high throughput: The next generation of Wi-Fi technology beyond 802.11 ax. IEEE Communications Magazine.

[ref-50] Lyu L, Yu H, Yang Q (2020). Threats to federated learning: a survey.

[ref-51] Ma C, Li J, Ding M, Yang HH, Shu F, Quek TQ, Poor HV (2020). On safeguarding privacy and security in the framework of federated learning. IEEE Network.

[ref-52] McMahan B, Moore E, Ramage D, Hampson S, y Arcas BA (2017). Communication-efficient learning of deep networks from decentralized data.

[ref-53] Mehrban S, Nadeem MW, Hussain M, Ahmed MM, Hakeem O, Saqib S, Kiah MM, Abbas F, Hassan M, Khan MA (2020). Towards secure FinTech: a survey, taxonomy, and open research challenges. IEEE Access.

[ref-54] Mothukuri V, Parizi RM, Pouriyeh S, Huang Y, Dehghantanha A, Srivastava G (2021). A survey on security and privacy of federated learning. Future Generation Computer Systems.

[ref-55] Naveed N, Anwar MN, Haq MIU (2021). Production and maintenance in industries: impact of industry 4.0. Industrial Robot: the International Journal of Robotics Research and Application.

[ref-56] Nilsson A, Smith S, Ulm G, Gustavsson E, Jirstrand M (2018). A performance evaluation of federated learning algorithms.

[ref-57] Nguyen DC, Ding M, Pathirana PN, Seneviratne A, Li J, Niyato D, Dobre O, Poor HV (2021b). 6G internet of things: a comprehensive survey. IEEE Internet of Things Journal.

[ref-58] Nguyen DC, Ding M, Pathirana PN, Seneviratne A, Li J, Poor HV (2021a). Federated learning for internet of things: a comprehensive survey. IEEE Communications Surveys & Tutorials.

[ref-59] Nguyen TD, Marchal S, Miettinen M, Fereidooni H, Asokan N, Sadeghi A-R (2019). DÏoT: a federated self-learning anomaly detection system for IoT.

[ref-60] Noura M, Atiquzzaman M, Gaedke M (2019). Interoperability in internet of things: Taxonomies and open challenges. Mobile networks and applications.

[ref-61] Pang J, Huang Y, Xie Z, Han Q, Cai Z (2020). Realizing the heterogeneity: a self-organized federated learning framework for IoT. IEEE Internet of Things Journal.

[ref-62] Parra GDLT, Rad P, Choo K-KR, Beebe N (2020). Detecting internet of things attacks using distributed deep learning. Journal of Network and Computer Applications.

[ref-63] Peltonen E, Bennis M, Capobianco M, Debbah M, Ding A, Gil-Castiñeira F, Jurmu M, Karvonen T, Kelanti M, Kliks A (2020). 6G white paper on edge intelligence.

[ref-64] Qian Y, Hu L, Chen J, Guan X, Hassan MM, Alelaiwi A (2019). Privacy-aware service placement for mobile edge computing *via* federated learning. Information Sciences.

[ref-65] Rahman SA, Tout H, Talhi C, Mourad A (2020). Internet of things intrusion detection: centralized, on-device, or federated learning?. IEEE Network.

[ref-66] Ray PP, Dash D, De D (2019). Edge computing for internet of things: a survey, e-healthcare case study and future direction. Journal of Network and Computer Applications.

[ref-67] Ren H, Deng J, Xie X (2022). Grnn: generative regression neural network—a data leakage attack for federated learning. ACM Transactions on Intelligent Systems and Technology (TIST).

[ref-68] Restuccia F (2021). IEEE 802.11 bf: toward ubiquitous Wi-Fi sensing.

[ref-69] Rizvi S, Kurtz A, Pfeffer J, Rizvi M (2018). Securing the internet of things (IoT): a security taxonomy for IoT.

[ref-70] Rothchild D, Panda A, Ullah E, Ivkin N, Stoica I, Braverman V, Gonzalez J, Arora R (2020). Fetchsgd: communication-efficient federated learning with sketching.

[ref-71] Saeed A, Ozcelebi T, Lukkien J (2019). Multi-task self-supervised learning for human activity detection. Proceedings of the ACM on Interactive, Mobile, Wearable, and Ubiquitous Technologies.

[ref-72] Safri H, Kandi MM, Miloudi Y, Bortolaso C, Trystram D, Desprez F (2022). A federated learning framework for IoT: application to industry 4.0.

[ref-73] Salim MM, Rathore S, Park JH (2020). Distributed denial of service attacks and its defenses in IoT: a survey. The Journal of Supercomputing.

[ref-74] Shaheen M, Farooq MS, Umer T, Kim B-S (2022). Applications of federated learning; taxonomy, challenges, and research trends. Electronics.

[ref-75] Sharma PK, Park JH, Cho K (2020). Blockchain and federated learning-based distributed computing defence framework for sustainable society. Sustainable Cities and Society.

[ref-76] Shen S, Zhu T, Wu D, Wang W, Zhou W (2022). From distributed machine learning to federated learning: in the view of data privacy and security. Concurrency and Computation: Practice and Experience.

[ref-77] Sikder AK, Petracca G, Aksu H, Jaeger T, Uluagac AS (2021). A survey on sensor-based threats and attacks to smart devices and applications. IEEE Communications Surveys & Tutorials.

[ref-78] Sodin D, Rudež U, Mihelin M, Smolnikar M, Čampa A (2021). Advanced edge-cloud computing framework for automated pmu-based fault localization in distribution networks. Applied Sciences.

[ref-79] Tang S, Shelden DR, Eastman CM, Pishdad-Bozorgi P, Gao X (2019). A review of building information modeling (BIM) and the internet of things (IoT) devices integration: present status and future trends. Automation in Construction.

[ref-80] Tran NH, Bao W, Zomaya A, Nguyen MN, Hong CS (2019). Federated learning over wireless networks: optimization model design and analysis.

[ref-81] Trindade S, Bittencourt LF, Da Fonseca NL (2022). Resource management at the network edge for federated learning. Digital Communications and Networks.

[ref-82] Tsukada M, Kondo M, Matsutani H (2020). A neural network-based on-device learning anomaly detector for edge devices. IEEE Transactions on Computers.

[ref-83] Unal D, Hammoudeh M, Khan MA, Abuarqoub A, Epiphaniou G, Hamila R (2021). Integration of federated machine learning and blockchain for the provision of secure big data analytics for Internet of Things. Computers & Security.

[ref-84] Victor N, Alazab M, Bhattacharya S, Magnusson S, Maddikunta PKR, Ramana K, Gadekallu TR (2022). Federated learning for IoUT: concepts, applications, challenges and opportunities.

[ref-85] Vijayakumar S, Dhasarathan N, Devabalan P, Jehan C (2019). Advancement and design of robotic manipulator control structures on cyber physical production system. Journal of Computational and Theoretical Nanoscience.

[ref-86] Wahab OA, Mourad A, Otrok H, Taleb T (2021). Federated machine learning: survey, multi-level classification, desirable criteria and future directions in communication and networking systems. IEEE Communications Surveys & Tutorials.

[ref-87] Wang G, Dang CX, Zhou Z (2019). Measure contribution of participants in federated learning.

[ref-88] Wang Y, Su Z, Zhang N, Xing R, Liu D, Luan TH, Shen X (2022). A survey on metaverse: fundamentals, security, and privacy. IEEE Communications Surveys & Tutorials.

[ref-89] Wang X, Wang C, Li X, Leung VC, Taleb T (2020). Federated deep reinforcement learning for Internet of Things with decentralized cooperative edge caching. IEEE Internet of Things Journal.

[ref-90] Xu Z, Yang Z, Xiong J, Yang J, Chen X (2019). Elfish: Resource-aware federated learning on heterogeneous edge devices. Ratio.

[ref-91] Yarradoddi S, Gadekallu TR (2022). Federated learning role in big data, jot services and applications security, privacy and trust in jot a survey. Trust, Security and Privacy for Big Data.

[ref-92] Yin X, Zhu Y, Hu J (2021). A comprehensive survey of privacy-preserving federated learning: a taxonomy, review, and future directions. ACM Computing Surveys (CSUR).

[ref-93] Zhang T, Gao L, He C, Zhang M, Krishnamachari B, Avestimehr AS (2022). Federated learning for the internet of things: applications, challenges, and opportunities. IEEE Internet of Things Magazine.

[ref-94] Zhang W, Yang D, Wu W, Peng H, Zhang N, Zhang H, Shen X (2021). Optimizing federated learning in distributed industrial IoT: a multi-agent approach. IEEE Journal on Selected Areas in Communications.

[ref-95] Zhao B, Fan K, Yang K, Wang Z, Li H, Yang Y (2021). Anonymous and privacy-preserving federated learning with industrial big data. IEEE Transactions on Industrial Informatics.

[ref-96] Zheng Z, Zhou Y, Sun Y, Wang Z, Liu B, Li K (2022). Applications of federated learning in smart cities: recent advances, taxonomy, and open challenges. Connection Science.

