# Supplementary material for: Integration of federated learning with IoT for smart cities applications, challenges, and solutions
Source: PeerJ Comput Sci. 2023 Dec 6;9:e1657. doi: 10.7717/peerj-cs.1657 (PMC10773731; doi:10.7717/peerj-cs.1657)
Supplement: Data S1 [file peerj-cs-09-1657-s001.docx]

## **Funding**

## This research received no external funding

## **Grant Disclosures**

## Not applicable.

## **Competing Interests**

The authors declare there are no competing interests.

## **Author Contributions**

Conceptualization, TM and I.H.; methodology, I.H.SFAS,; software, TM,I.H., and T.M.; validations FAS. and KO; formal analysis, I.H, T.M.; investigation, I.H, T.M.,YG; resources, YG and I.H; Data duration, I.H, YG, and HH Writing—original draft preparation, TM,KO and HH.; Writing—review and editing, I.H.;T.M and KO. Visualization, I.H. and TM

## **Data Availability**

Not available.

## **Supplemental Information**

Not applicable.

.
